# Nanoparticle-Enriched Sodium Fluoride Gel with and Without Er, Cr: YSGG Laser Activation: Effects on Enamel Microhardness and Sealant Bond Performance on Demineralized Enamel

**DOI:** 10.3390/gels12070597

**Published:** 2026-07-03

**Authors:** Mohammed A. Alrabiah, Fahad Alkhudhairy

**Affiliations:** 1 Department of Prosthetic Dental Science, College of Dentistry, King Saud University, Riyadh 11545, Saudi Arabia; mohalrabiah@ksu.edu.sa; 2 Restorative Dental Sciences Department, College of Dentistry, King Saud University, Riyadh 11545, Saudi Arabia

**Keywords:** sodium fluoride gel, bioactive glass nanoparticles, Er, Cr: YSGG laser, hydroxyapatite nanoparticles, microhardness, pit and fissure sealants

## Abstract

This study aimed to assess the remineralization efficacy of NaF gel enriched with hydroxyapatite nanoparticles (HANPs) and bioactive glass nanoparticles (BAGNPs), with and without adjunctive Er, Cr: YSGG laser irradiation (ECL; 0.5 W, 5 Hz, 20 mJ/pulse, 60 µs pulse duration, water–air spray), on artificially demineralized enamel by evaluating enamel microhardness (MH), resin tag length (RTL), and shear bond strength (SBS) of pit and fissure sealants (PFSs). A total of 168 extracted human third molars free from cracks, fractures, erosion, enamel hypoplasia, surface irregularities, and any history of prior chemical or fluoride treatment were included in the study. All samples underwent continuous immersion in a demineralizing solution until specific DIAGNOdent values of 10–25 were achieved. Samples were randomly allocated into six groups (*n* = 28): Group 1 (untreated control), Group 2 (NaF gel), Group 3 (NaF + HANPs), Group 4 (NaF + BAGNPs), Group 5 (NaF + HANPs-ECL), and Group 6 (NaF + BAGNPs-ECL). Enamel MH was assessed using a Vickers MH tester (*n* = 8). RTL was evaluated using scanning electron microscopy (SEM) (*n* = 8). SBS was measured using a universal testing machine (*n* = 12), followed by failure mode analysis. Data were analyzed using *ANOVA* and *Tukey’s post hoc* test (*p* < 0.05). Group 5 (NaF + HANPs-ECL) exhibited the highest values for MH (366.20 ± 26.11 HV), RTL (70.34 ± 2.57 µm), and SBS (13.67 ± 0.35 MPa), whereas the untreated control group exhibited the lowest values for all the outcomes. Groups 1 and 2 demonstrated comparable RTL and SBS values (*p* > 0.05). The remaining groups exhibited significantly different MH, RTL, and SBS values (*p* < 0.05). The ECL-assisted nanoparticle-integrated NaF gel significantly enhanced enamel MH, RTL, and shear SBS of PFS compared to NaF gel alone. HANPs demonstrated superior remineralization outcomes compared to BAGNPs across all tested parameters. The present findings support the adjunctive use of laser activation with nanoparticle-modified NaF gel as a promising strategy for optimizing sealant performance on demineralized enamel.

## 1. Introduction

Oral diseases represent a significant global health burden, exerting a profound impact across all stages of life through pain, functional impairment, and aesthetic compromise. According to the Global Burden of Disease Study 2017, approximately 3.5 billion individuals worldwide are affected by oral conditions, with caries of the permanent dentition being the most prevalent pathological entity [1]. White spot lesions (WSLs) are the earliest clinically visible signs of enamel demineralization and mark the initial stage of caries disease. If left unaddressed, the progressive dissolution of the mineral phase culminates in cavitation [2,3]. Pit and fissure sealants (PFSs) have been widely advocated as a primary caries-preventive modality, with resin-based sealants (RBSs) and glass ionomer (GI) being the principal materials employed in clinical practice [4,5]. However, the adhesive performance of RBS is substantially compromised when applied to demineralized enamel, as the mineral-deficient substrate lacks the inorganic crystallite architecture required for reliable micromechanical interlocking with the sealant resin [6]. Therefore, remineralizing agents must be applied to WSLs before sealant placement.

Fluoride is an effective remineralizing agent for enamel and can arrest the formation and progression of WSLs [7,8]. However, topical fluoride application before acid etching has been shown to reduce the bond strength of PFS, leading to the clinical recommendation that sealant placement precede fluoride therapy or be deferred to a separate session [9,10]. Moreover, the remineralizing efficacy of fluoride remains limited to the outermost 10–30 µm of the enamel lesion, rendering it insufficient for restoring deeper subsurface mineral loss [11].

Biomimetic approaches have been employed in recent years to develop nanomaterials for the remineralization of early enamel lesions. To overcome these challenges, caries management research is shifting toward multifunctional innovations [12]. Nanoparticles in restorative dentistry are revolutionizing treatments by improving material strength and wear resistance while introducing antibacterial properties [13,14]. Asadi et al. demonstrated that supplementing sodium fluoride (NaF) gel with various nanoparticles significantly improved subsurface enamel remineralization, yielding promising outcomes that support the potential clinical utility of this combined approach [15]. The application of hydroxyapatite nanoparticles (HANPs) for the repair of early enamel lesions has received considerable attention [16]. They serve as rich sources of calcium and phosphate ions, which are essential for remineralizing demineralized enamel and dentin [17]. The results of a study conducted by Xiangcai et al. showed that the greater the amount of HANP in the NaF mouthrinse, the greater the enamel remineralization [18]. Similarly, bioactive glass nanoparticles (BAGNPs) have attracted considerable attention for their role in dentin remineralization, primarily through the deposition of calcium phosphate on their surfaces [19]. Owing to their high calcium content and strong bonding to hard tissues, BAGNPs are widely used in bone regeneration and management of dentin hypersensitivity [20,21]. BAG facilitates apatite formation, improving the radiodensity and MH of demineralized dentin. Wu et al. reported that the remineralization zone produced by BAG is significantly greater than that achieved with CPP-ACP and fluoride [22]. Existing evidence confirms that individual remineralizing agents—fluoride, HANPs, BAGNPs, and Er, Cr: YSGG laser—each improve enamel mineral density to varying degrees. However, a critical and clinically unresolved question remains: does the degree of mineral recovery achievable by these agents translate into functionally superior adhesive performance of pit and fissure sealants on demineralized enamel? Mineral density alone does not ensure adequate resin micromechanical interlocking, and no prior study has systematically characterized the relationship between nanoparticle-mediated remineralization—with or without laser activation—and the downstream RTL and SBS of sealants bonded to such substrates. This represents the operative clinical gap that the present study was designed to address.

The introduction of lasers into dentistry by Maiman in the 1960s paved the way for their expanded application across various dental disciplines [23]. Notably, Maddah et al. reported that Er, Cr: YSGG laser (ECL) irradiation synergistically promotes enamel remineralization when combined with HANPs [24]. Similarly, Klarić et al. evaluated the Er, Cr: YSGG laser on BAG on human enamel subjected to radiotherapy. The combination treatment demonstrated a remineralization effect on the surface of irradiated enamel, as evidenced by an increase in MH [24]. However, the combined approach of a NaF gel enriched with HANPs and BAG NPs activated by an Er, Cr: YSGG laser on RTL, MH, and SBS remains limited. The present study was conducted under two null hypotheses: (H_01_) there would be no statistically significant difference in enamel MH among specimens remineralized using (HANP, BAG alone in NAF gel and HANP, BAG in NAF gel activated by Er, Cr: YSGG laser) compared to demineralized control; and (H_02_) the RTL and SBS of PFS bonded to enamel remineralized using (HANP, BG alone in NAF gel and HANP, BAG in NAF gel activated by Er, Cr: YSGG laser) would be comparable to the demineralized control group. Therefore, this study aimed to determine whether the degree of mineral recovery achievable by nanoparticle-modified NaF gel, with and without ECL irradiation, is sufficient to restore the structural substrate required for reliable sealant adhesion—a translational question that prior investigations have not systematically addressed.

## 2. Results

**Figure 1:**
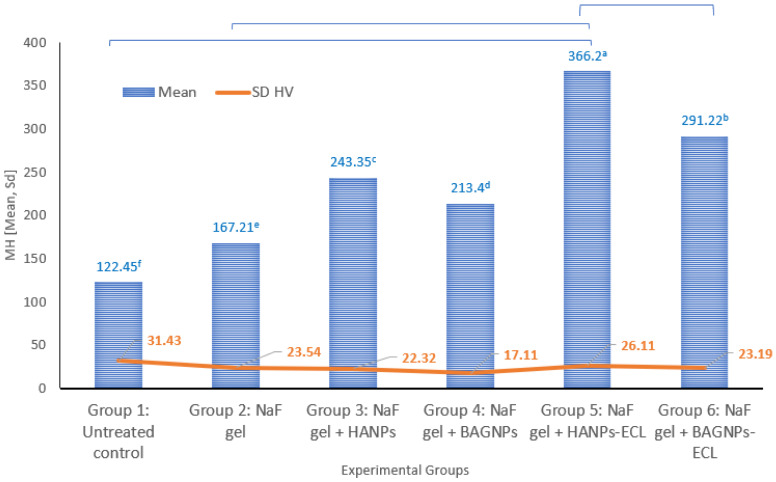
ANOVA analysis showing individual enamel MH values with mean ± SD across experimental groups. *Mean ± standard deviation. Different superscript letters indicate statistically significant intergroup differences (Tukey HSD post hoc test, p < 0.05). ECL = Er, Cr: YSGG laser; HANPs = hydroxyapatite nanoparticles; BAGNPs = bioactive glass nanoparticles*.

MH evaluation: Figure 1 displays the MH assessment of the enamel after using different remineralizing agents. The highest MH value was recorded in Group 5 (NaF gel + HANPs-ECL: 366.20 ± 26.11 HV), whereas the lowest was observed in Group 1 (untreated control: 122.45 ± 31.43 HV). Intergroup analysis revealed that all groups demonstrated significantly different MH outcomes (*p* < 0.05). The overall descending rank order of microhardness was Group 5 > Group 6 (NaF + BAGNPs-ECL: 291.22 ± 23.19 HV) > Group 3 (NaF + HANPs: 243.35 ± 22.32 HV) > Group 4 (NaF + BAGNPs: 213.40 ± 17.11 HV) > Group 2 (NaF gel: 167.21 ± 23.54 HV) > Group 1. One-way ANOVA revealed a statistically significant effect of remineralizing treatment on enamel MH (F(5, 42) = 201.68, *p* < 0.001; η^2^ = 0.96), indicating that 96% of the total variance in MH was attributable to the treatment applied. Comparable analyses for RTL (F(5, 42) = 271.60, *p* < 0.001; η^2^ = 0.97) and SBS (F (5, 66) = 206.82, *p* < 0.001; η^2^ = 0.94) confirmed large treatment effects across all three outcome measures (all η^2^ > 0.14 by conventional benchmarks).

**Figure 2:**
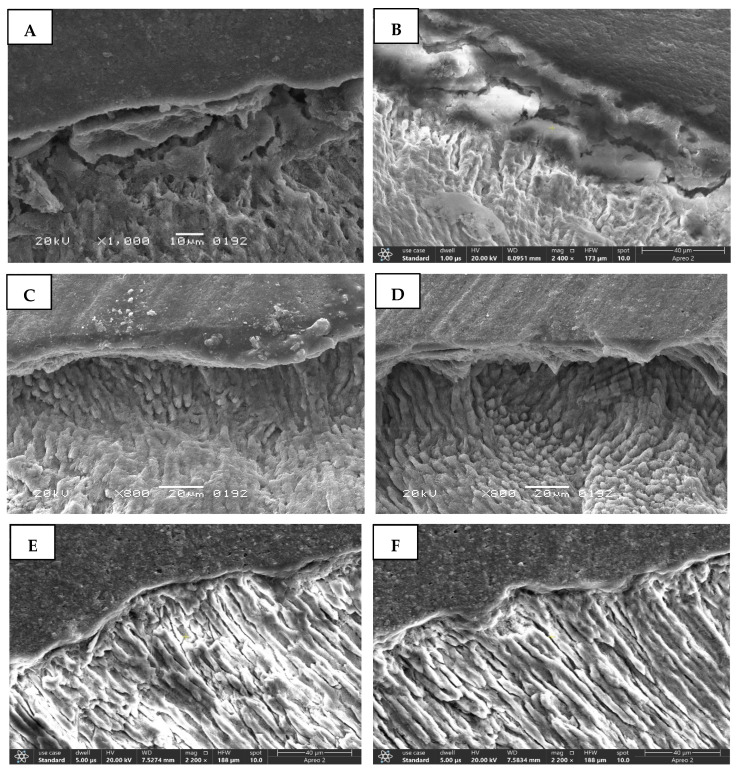
(**A**) SEM micrograph showing a relatively intact, dense superficial enamel layer, consistent with the characteristic “surface zone” preservation. Immediately beneath the surface zone, extensive demineralization is evident. The enamel microstructure appears highly porous, irregular, and roughened, with deep inter-prismatic dissolution channels. (**B**) SEM image of enamel remineralized with NaF gel. A thin, relatively continuous surface layer is present at the top, consistent with fluorapatite formation. Scattered bright micro-deposits are visible across the surface (consistent with CaF_2_ globule formation), a hallmark of topical fluoride application. (**C**) SEM micrograph of NaF gel + HANPs-treated specimens shows a well-defined, continuous surface layer with dense globular mineral deposits and a significant reduction in inter-prismatic void spaces. In contrast, (**D**) SEM micrograph of NaF gel + BAGNPs-treated specimens shows a moderately dense surface layer with characteristically angular mineral deposits and partial occlusion of subsurface porosities, consistent with bioactive glass ion-exchange-mediated calcium phosphate precipitation. (**E**,**F**) SEM micrograph of NaF gel + BAGNPs and NaF gel + HANPs activated by Er, Cr: YSGG, characterized by near-complete obliteration of inter-prismatic voids and tightly packed elongated mineral columns oriented perpendicular to the enamel surface, reflecting extensive mineral infilling of inter-prismatic spaces, with well-organized elongated crystallite columns, consistent with ECL irradiation-associated enhancement of mineral organization irrespective of nanoparticle type, as inferred from morphological features alone.

RTL evaluation—Table 1 displays the RTL assessment of pits and fissure sealants bonded to enamel after using different remineralizing agents. The longest resin tags were recorded in Group 5 (NaF gel + HANPs-ECL: 70.34 ± 2.57 µm; visual score 3), whereas the shortest RTL was observed in Group 1 (untreated control: 32.65 ± 3.54 µm; visual score 1). Intergroup comparisons revealed no statistically significant difference in RTL between Group 1 (32.65 ± 3.54 µm) and Group 2 (31.24 ± 2.23 µm), with both groups recording a visual score of 1 (*p* > 0.05). However, the remaining groups (Group 3 (NaF + HANPs: 58.12 ± 2.43 µm; visual score 2), Group 4 (NaF + BAGNPs: 52.19 ± 2.32 µm; visual score 2), Group 5, and Group 6 (NaF + BAGNPs-ECL: 64.21 ± 3.11 µm; visual score 3) exhibited significantly different outcomes from one another (*p* < 0.05) (Figure 2A–F).

**Figure 3:**
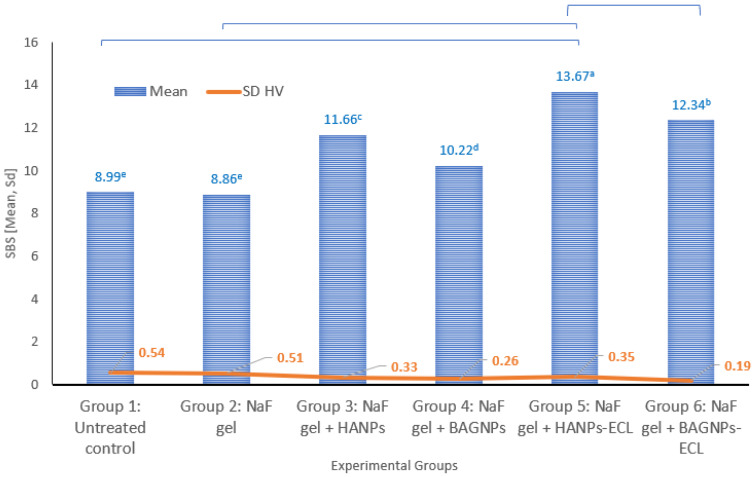
ANOVA analysis showing individual shear bond strength (SBS) values (MPa) with mean ± SD across experimental groups. *Mean ± standard deviation. Different superscript letters (a–e) above data points indicate statistically significant intergroup differences (Tukey HSD post hoc test, p < 0.05). Groups 1 and 2 share a superscript “e”, indicating no significant difference between them. ECL = Er, Cr: YSGG laser; HANPs = hydroxyapatite nanoparticles; BAGNPs = bioactive glass nanoparticles*.

SBS outcomes—Figure 3 displays the shear bond strength of pit and fissure sealants bonded to enamel after using different remineralizing agents. The highest SBS value was recorded in Group 5 (NaF gel + HANPs-ECL: 13.67 ± 0.35 MPa), whereas the lowest was observed in Group 2 (NaF gel: 8.86 ± 0.51 MPa). Intergroup comparisons revealed no statistically significant difference in SBS between Group 1 (untreated control: 8.99 ± 0.54 MPa) and Group 2 (NaF gel: 8.86 ± 0.51 MPa), as both groups shared the same superscript, indicating comparable bonding outcomes (*p* > 0.05). In contrast, the remaining groups showed significantly different SBS values (*p* < 0.05). The overall descending rank order of shear bond strength was Group 5 > Group 6 (NaF gel + BAGNPs-ECL: 12.34 ± 0.19 MPa) > Group 3 (NaF gel + HANPs: 11.66 ± 0.33 MPa) > Group 4 (NaF gel + BAGNPs: 10.22 ± 0.26 MPa) > Group 1 > Group 2. Pearson correlation analysis across all specimens revealed strong positive associations between MH and SBS (r = 0.96, *p* < 0.001), MH and RTL (r = 0.97, *p* < 0.001), and RTL and SBS (r = 0.95, *p* < 0.001), collectively confirming that the degree of mineral recovery was a robust determinant of downstream sealant adhesive performance. These correlations directly substantiate the study’s central hypothesis that a functional remineralization threshold governs sealant bonding outcomes on demineralized enamel.

**Figure 4:**
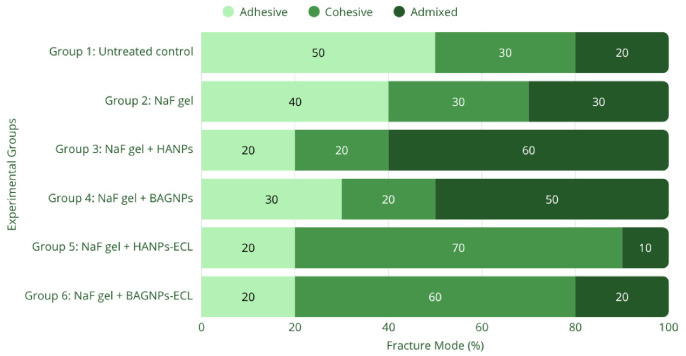
Each stacked bar represents 100% of failure modes observed per group. Cohesive failure (teal) is the most favorable mode, indicating failure within the material rather than at the enamel–sealant interface. Adhesive failure (blue) is the least favorable, reflecting interfacial bond weakness. Admixed failure (amber) represents a combination of both modes. ECL = Er, Cr: YSGG laser; HANPs = hydroxyapatite nanoparticles; BAGNPs = bioactive glass nanoparticles.

Fracture pattern analysis—Figure 4 presents the evaluation of fracture patterns across all tested groups. Cohesive fractures were most frequently observed in both laser-assisted groups. Samples treated with NaF-HANPs and NaF-BAGNPs showed predominantly admixed fracture patterns. In contrast, the NaF-treated and control groups mainly exhibited adhesive failures.

### Discussion

The present study was conducted under two null hypotheses: (H_01_) there would be no statistically significant difference in enamel MH among specimens remineralized using NaF gel containing HANPs and BAGNPs, with and without ECL irradiation, compared to demineralized controls; and (H_02_) RTL and SBS of PFS bonded to remineralized enamel would be comparable to those of controls. H_01_ was rejected, as all experimental groups exhibited significantly greater MH than demineralized controls. H_02_ was partially rejected, as RTL and SBS in the NaF-gel-only group were statistically comparable to the untreated control. The concurrent use of MH testing, SEM, RTL measurement, and SBS analysis was intentional; this multimodal approach ensured that conclusions regarding remineralization efficacy were supported by convergent mechanical and morphological evidence, enhancing the translational relevance of the findings [15]. Collectively, the results provide the first systematic evidence that the degree of mineral recovery—and not merely its presence—determines sealant adhesive outcomes on demineralized enamel, establishing a functional threshold concept for clinically meaningful remineralization before sealant placement.

The NaF gel + HANPs-ECL group exhibited the highest MH, followed by NaF gel + BAGNPs-ECL, consistent with the report of Maddah et al. that ECL irradiation significantly enhances remineralization by promoting HANP uptake into the demineralized enamel surface [24]. The superior MH outcomes in the laser-assisted groups are consistent with well-characterized thermal and physicochemical modifications attributed to ECL irradiation in the prior literature, including changes proposed to involve hydroxyapatite crystal reorganization, increased crystal size and mineral density, reduction in inter-prismatic porosity, and depletion of carbonate, water, and organic components within the enamel matrix [25,26,27]; direct compositional verification of these changes was not performed in the present study. These modifications are reported to produce a fused, less acid-soluble surface layer with greater structural resistance than that achievable through remineralizing agent application alone [28]. At the sub-ablative parameters employed (0.5 W, 5 Hz, 60 µs, non-contact at 5 mm), Er, Cr: YSGG irradiation at 2780 nm may additionally facilitate nanoparticle infiltration into enamel microporosities via photomechanical shock, and residual thermal energy may promote subsurface mineral reorganization; these mechanisms are not mutually exclusive and likely act synergistically, though their relative contributions cannot be partitioned without dedicated laser-only control groups [26,29,30,31,32,33]. Accordingly, a portion of the MH and RTL gains observed in Groups 5 and 6 may reflect the laser’s independent physicochemical contribution to enamel surface modification, which remains unquantified in the present dataset and warrants cautious interpretation.

Among the non-laser groups, HANP-treated specimens exhibited significantly greater MH than BAGNPs- and NaF-gel-only groups. This corroborates the findings of Imran et al., who reported that nano-hydroxyapatite infiltrates sub-surface enamel porosities and is proposed to act as a template supporting calcium phosphate mineral deposition, as inferred from microhardness and compositional data in that study; direct crystallographic verification was not performed in the present work [34]. BAGNPs demonstrated intermediate remineralizing performance, consistent with the SEM-based findings of Sebastian et al., who reported that BAGNPs occlude demineralization-induced fissures with deposits exhibiting morphology consistent with bioactive glass crystallization kinetics [35]. This behavior is attributed to the ion-exchange capacity of bioactive glass, whereby calcium, phosphate, and silicate ion release is proposed to promote formation of a calcium phosphate-like mineral layer analogous to carbonated hydroxyapatite, as characterised by EDX and XRD in the prior literature; mineral composition was not directly verified in the present study [36,37]. NaF gel yielded significantly greater MH than demineralized controls but remained inferior to all other experimental agents. This is consistent with the dual remineralization pathways attributed to fluoride in the prior literature—proposed substitution of hydroxyl groups within the hydroxyapatite lattice to generate fluorapatite, and concurrent deposition of calcium fluoride globules on the enamel surface—neither of which was directly verified in the present study, as no EDX, XRD, or XPS analysis was performed; these mechanistic attributions therefore reflect established literature-based interpretations rather than outcomes confirmed herein [38,39].

Laser-assisted HANP- and BAGNP-pretreated specimens displayed the longest resin tags and highest SBS values, consistent with the findings of Al-Qahtani et al., who reported that Er, Cr: YSGG-activated NaF gel produced the highest MH and micro-shear bond strength among composite restorations bonded to demineralized dentin [40]. At sub-ablative parameters, laser irradiation may further modify the enamel surface through thermally driven removal of organic matrix components and desiccation of existing microporosities, potentially facilitating deeper resin tag penetration; whether this reflects synergistic interaction with the nanoparticle phase or parallel independent contributions cannot be resolved without laser-only control groups [41,42]. Subsequent infiltration of HANPs and BAGNPs into conditioned porosities is consistent with mineral phase deposition within the enamel microstructure, as inferred from the microhardness gains observed in this and prior research; direct compositional confirmation was not performed in the present study. This is proposed to reinforce the surface layer and create a mineral-rich substrate with greater cohesive strength, thereby reducing the risk of adhesive failure at the enamel–resin interface [43]. Conversely, NaF gel treatment yielded RTL and SBS values comparable to those of the untreated control, suggesting that fluoride application alone was insufficient to meaningfully enhance sealant-enamel bonding. This may be attributable to the conversion of surface hydroxyapatite into fluorapatite, which—despite conferring greater acid resistance—may render the enamel surface less susceptible to phosphoric acid etching, thereby compromising microporosity formation required for resin micromechanical interlocking [9]. Additionally, NaF gel may deposit a surface film that impedes resin infiltration and limits sealant penetration depth [44].

The laser-assisted groups predominantly exhibited cohesive fracture patterns, whereas NaF gel + HANPs and NaF gel + BAGNPs groups displayed admixed failure distributions, and adhesive failures predominated in the NaF-gel-only and untreated control groups. These fracture mode distributions are consistent with and further corroborate the SBS hierarchy observed across all groups. Although high-magnification fractographic analysis (≥2000×) was not performed—precluding definitive discrimination between sealant-cohesive and enamel-cohesive fracture modes—sealant-cohesive fracture remains the most parsimonious interpretation on three grounds: the substantially restored MH values in Groups 5 and 6 are mechanically inconsistent with a weakened surface layer; DIAGNOdent-guided demineralization to values of 10–25 preserved the structural integrity of the underlying enamel bulk; and the peak shear forces recorded (≤13.67 MPa) fall well below the cohesive fracture threshold of resin-based sealants, collectively rendering enamel-bulk fracture physically improbable at the forces employed.

A potential limitation of this study is that the in vitro conditions may not fully replicate the complexity of the clinical oral environment. Artificial demineralization produces chemically uniform lesions that may not fully capture the heterogeneous biofilm-mediated mineral loss of naturally occurring caries; moreover, dynamic oral variables—including salivary flow, thermal cycling, masticatory loading, and microbial challenge—were not simulated in the present model. The single nanoparticle concentration, application duration, and laser output settings evaluated herein represent a starting point rather than an optimized clinical protocol; dose–response relationships across varying concentrations, application frequencies, and laser parameters must be established before clinical translation can be recommended. Direct compositional verification of the remineralized layers was not performed, as EDX spectroscopy, XPS, Raman spectroscopy, and cross-sectional TEM were not employed; future studies should incorporate these modalities alongside AFM-based surface topography analysis to provide mechanistic confirmation of the mineral phase changes inferred from the surrogate outcomes used herein. Additionally, high-magnification SEM fractographic analysis (≥2000×) was not performed, precluding definitive discrimination between sealant-cohesive and enamel-cohesive fracture modes in the laser-assisted groups [25,45]. Although direct thermocouple measurement at the dentin-enamel junction was not performed, the thermal safety of the parameters employed (0.5 W, 5 Hz, 20 mJ/pulse) is supported by established biophysical evidence: at 2780 nm, Er, Cr: YSGG energy is absorbed predominantly by superficial enamel water (absorption coefficient ~4,000 cm^−1^), confining energy deposition to the outermost enamel with negligible conductive penetration to dentinal structures [46]; incident energy at sub-ablative fluences is dissipated primarily through evaporative cooling of the water–air spray rather than conductive heat transfer [29]; and Rizoiu et al. reported intrapulpal temperature rises of less than 1.5 °C under comparable Er, Cr: YSGG settings with water–air spray, remaining well within the critical pulpal threshold of 5.5 °C above baseline [47]. The 200 ms inter-pulse interval at 5 Hz substantially exceeds enamel thermal relaxation time (~1–3 ms), precluding cumulative heat build-up between successive pulses. Furthermore, the absence of a dedicated laser-only control group precludes isolation of the laser’s independent contribution to microporosity and enamel surface modification from that of nanoparticle supplementation; a factorial 2 × 2 design incorporating laser-only and nanoparticle-only arms would be required to partition these contributions definitively. Baseline MH of sound enamel was not recorded before demineralization, precluding calculation of percentage surface microhardness recovery (%SMHR); future studies should incorporate pre-demineralization MH measurements to enable normalization against individual specimen baselines. Finally, the present study’s distinct contribution lies not in novel material combinations per se, but in the characterization of their functional adhesive consequences—an outcome domain that prior investigations have not systematically addressed.

## 3. Conclusions

The adjunctive use of ECL irradiation in conjunction with nanoparticle-integrated sodium fluoride gel significantly enhanced enamel microhardness, resin tag length, and shear bond strength of pit and fissure sealants compared to sodium fluoride gel alone or untreated demineralized enamel. Hydroxyapatite nanoparticles demonstrated superior remineralization outcomes compared to bioactive glass nanoparticles across all tested parameters. These observed performance outcomes support the adjunctive use of laser irradiation with nanoparticle-modified fluoride agents as a promising clinical strategy to optimize sealant performance on demineralized enamel, pending mechanistic confirmation through factorial designs incorporating laser-only control arms.

## 4. Materials and Methods

Sample Selection and Sample Size Calculation: One hundred sixty-eight extracted human third molars were obtained from adult patients aged 18–30 years following written informed consent. Teeth were screened before inclusion, and samples free from cracks, fractures, erosion, enamel hypoplasia, surface irregularities, or any history of prior chemical or fluoride treatment were included. All teeth were extracted for orthodontic or third molar impaction-related indications, excluding pathological extractions, to minimize the risk of pre-existing enamel alterations that were undetectable by visual inspection alone. The exclusion criteria were verified independently by two calibrated examiners under standardized magnification before inclusion. The sample size was determined using the WHO sample size calculation. Using WHO sample size calculator in Health Studies [48], for estimating sample size in ANOVA design with six groups, taking a confidence level of 95%, required absolute precision of 1%, mean of remineralizing in the pretreatment group of 2.24 + 0.08 [49], and variance of 0.0064, The minimum required sample size was 28 specimens per group, which was adopted as the final allocation across all six groups, yielding a total sample size of 168 teeth [5,49]. Immediately after extraction, residual soft tissue and calculus deposits were removed from the tooth surfaces using a hand scaler (Hu-Friedy, Chicago, IL, USA). Samples were disinfected by immersion in a 0.1% chloramine-T solution (Sigma-Aldrich, St. Louis, MO, USA) for one week post-surgery. Following disinfection, all teeth were thoroughly rinsed and stored in fresh distilled water at 37 °C in a temperature-controlled incubator (Memmert GmbH, Schwabach, Germany) until use. All specimens were used within one month of extraction to minimize post-extraction alterations in the enamel composition and mechanical properties. Each tooth was subsequently mounted vertically in a self-curing acrylic resin (DPI RR Cold Cure, Dental Products of India, Mumbai, India) up to the cementoenamel junction. Cuspal surfaces were uniformly flattened using a flat-head diamond bur (KG Sorensen, São Paulo, Brazil) under copious water irrigation, removing approximately 1 mm of enamel to generate a planar occlusal surface comprising solely enamel, confirmed by the absence of dentinal color change and probe-based depth measurement [5,50].

Demineralization and pH Cycling—Artificial enamel lesions were induced by daily immersion of each specimen in a freshly prepared demineralizing solution (2.2 mM CaCl_2_, 2.2 mM NaH_2_PO_4_·2H_2_O, 0.05 M acetic acid; pH 4.4 adjusted with 1 M KOH). Lesion progression was monitored daily using DIAGNOdent™ (KaVo, Biberach, Germany), and demineralization was discontinued once readings of 10–25 (consistent with superficial enamel caries) were recorded [51]. Following demineralization, all specimens were rinsed and stored in deionized water and allocated into six groups (*n* = 28) based on the assigned remineralizing intervention.

**Group 1:** No remineralizing agent was applied to the enamel.

**Group 2:** NaF gel-1.1% NaF gel (Flor-Opal, Ultradent Products Inc., South Jordan, UT, USA) was applied to the demineralized enamel surface and left undisturbed for 120 s, after which the excess was removed by rinsing with distilled water for 15 s, followed by gentle air drying [52].

**Group 3:** NaF gel + HANPs: HANPs (Sigma Aldrich, Berlin, Germany) were incorporated into 1.1% NaF gel by first preparing a homogeneous nanoparticle suspension. A predetermined quantity of HANPs (typically 5 wt.%) was dispersed in distilled water and subjected to ultrasonication for 15–30 min to minimize agglomeration and ensure uniform distribution. This suspension was gradually added to the NaF gel under continuous magnetic stirring to achieve proper mixing. A small number of surfactants, such as Tween 80, were added to improve stability and prevent particle aggregation. The final gel was adjusted to a near-neutral pH (approximately 7) to maintain both fluoride efficacy and hydroxyapatite stability and stored in airtight, light-resistant containers until use. The mixture was then applied to the WSL and left for 2 min. Afterward, the surface was rinsed and air-dried [53,54].

**Group 4:** NaF gel + BAGNPs-5 wt% BAGNPs (Sigma Aldrich, Berlin, Germany) were incorporated into 1.1% NaF gel following the same dispersion and homogenization protocol as described for Group 3. The resultant formulation was subsequently applied to the demineralized enamel surface using the same procedure outlined for Group 3 [55].

**Group 5:** HANPs-ECL + NaF gel–HANPs-modified 1.1% NaF gel was applied to the demineralized enamel surface in a manner identical to that described for Group 3. The treated surface was then irradiated with an Er, Cr: YSGG laser (Waterlase iPlus, BIOLASE Inc., Foothill Ranch, CA, USA) at 2780 nm. The laser parameters were configured as follows: power of 0.5 W, pulse energy of 20 mJ, pulse duration of 60 µs, and repetition frequency 5 Hz, with a concurrent water–air coolant spray maintained at a 60%:40% ratio. The samples were irradiated for a total of 20 s using a gold handpiece (MZ6) equipped with a sapphire tip measuring 600 µm in diameter and 4 mm in length. Throughout irradiation, the handpiece was oriented perpendicularly to the enamel surface at a fixed non-contact distance of 5 mm and advanced in a sweeping motion to achieve homogeneous energy delivery across the treated area [56,57].

**Group 6:** NaF gel + BAGNPs-ECL-The BAGNPs-modified NaF gel was applied to the demineralized enamel surface following the protocol described for Group 3. The treated surface was subsequently subjected to Er, Cr: YSGG laser irradiation using the same parameters and technique as those outlined for Group 5.

Following remineralization, all samples were stored in artificial saliva (Nanochemazone, New Delhi, India) until further use.

Microhardness (MH) measurement (*n* = 8)—MH measurements were performed on eight samples from each cohort using a Vickers microhardness tester (HVS-100 Digital Display Hardness Tester, Jinan Sanyun Testing Instrument Co., Ltd., Jinan, China) under a standardized load of 10 N, with a dwell time of 10 s. Three indentations were made on each specimen at 1 mm intervals, and the mean of the three readings was recorded as the final MH value for each specimen [58,59].

Bonding of PFS—Following completion of the remineralization protocols, all enamel surfaces were etched with 37% phosphoric acid gel (ScotchBond Etchant, 3M ESPE, St. Paul, MN, USA) for 20 s, thoroughly rinsed with distilled water, and air-dried for 10 s using an oil-free air spray. A resin-based PFS (Clinpro™, 3M ESPE, St. Paul, MN, USA) was applied to the etched enamel surface in accordance with the manufacturer’s instructions and light cured for 20 s using an LED curing light (Elipar™ DeepCure-S, 3M ESPE, St. Paul, MN, USA) at a light intensity of ≥1000 mW/cm^2^ to ensure complete polymerization of the sealant material [60].

**SEM analysis:** Eight specimens from each experimental group were selected and longitudinally sectioned through the midline using a low-speed diamond saw to expose the sealant–enamel interface. The sectioned surfaces were ultrasonically cleaned, dehydrated through an ascending series of ethanol concentrations, and sputter-coated with a gold-palladium alloy (Sputter Coater SC7620; Quorum Technologies, East Sussex, UK) to ensure adequate surface conductivity before imaging. Specimens were subsequently examined under a scanning electron microscope (Topcon ABT 150S, Topcon Co., Tokyo, Japan) at standardized magnifications to assess RTL and the interfacial morphology. RTL was measured in micrometers (µm) at three representative sites per specimen using the microscope’s built-in calibrated measurement software, and the mean value was recorded for each specimen [5]. Qualitative evaluation of the resin–enamel interface was performed using the four-step visual scoring system (scores 0–3) described by Ferrari et al. [61]. All SEM evaluations and visual scoring were performed independently by two calibrated examiners who were blinded to the group allocation to eliminate assessment bias. Intra-examiner reliability was determined using Cohen’s kappa coefficient, which yielded a value of 0.87, indicating a strong agreement between the examiners.

Shear Bond Strength (SBS) Assessment—SBS at the enamel–sealant interface was evaluated using a universal testing machine (Instron 3365; Instron Corp., Norwood, MA, USA). Each specimen was securely mounted in the testing jig and subjected to a shear load applied using a straight knife-edge chisel-shaped rod positioned at the enamel–sealant interface parallel to the bonded surface. A crosshead speed of 0.5 mm/min was maintained under a maximum load cell capacity of 100 N until cohesive, adhesive, or admixed failure of the bonded interface was observed. The SBS value for each specimen was calculated by dividing the load at failure (N) by the bonded surface area (mm^2^) and expressed in megapascals (MPa) using the following formula:

SBS (MPa) = Force at failure (N) ÷ Bonded surface area (mm^2^)

Following debonding, all specimens were examined under a stereomicroscope (Leica S9i; Leica Microsystems GmbH, Wetzlar, Germany) at 40× magnification to determine the fracture mode. Failure patterns were classified into three categories: adhesive, cohesive, and admixed [49].

**Statistical Analysis:** Data were analyzed using the Statistical Package for the Social Sciences (SPSS) version 29.0 (IBM Corp., Armonk, NY, USA). The Shapiro–Wilk test was used to assess data normality. *ANOVA* and *Tukey post hoc* tests were used to compare the MH, RTL, and SBS (*p* < 0.05). Effect size was quantified using η^2^ for each one-way ANOVA, with thresholds of 0.01, 0.06, and 0.14 interpreted as small, medium, and large effects, respectively. Pearson bivariate correlation analysis was conducted across all specimens to quantify the relationships between MH, RTL, and SBS outcomes, in order to statistically characterize the association between the degree of mineral recovery and downstream sealant adhesive performance. We computed 95% confidence intervals for group means of all three outcome measures and report these alongside descriptive statistics to facilitate interpretation of the precision of effect estimates.

## Figures and Tables

**Table 1:** RTL assessment of pit and fissure sealants bonded to enamel after using different Remineralizing Agents.

Remineralizing Agents	Visual Score	Mean ± SD (μm)	95% CI (μm)	*p*-Value †
Group 1: Untreated control	1	32.65 ± 3.54 **^e^**	29.69–35.61	***p* < 0.05**
Group 2: NaF gel	1	31.24 ± 2.23 **^e^**	29.38–33.10	
Group 3: NaF gel + HANPs	2	58.12 ± 2.43 **^c^**	56.09–60.15	
Group 4: NaF gel + BAGNPs	2	52.19 ± 2.32 **^d^**	50.25–54.13	
Group 5: NaF gel + HANPs-ECL	3	70.34 ± 2.57 **^a^**	68.19–72.49	
Group 6: NaF gel + BAGNPs-ECL	3	64.21 ± 3.11 **^b^**	61.61–66.81	

† ANOVA, *p* < 0.05. Distinct superscripts (a–e) denote significant intergroup differences (Tukey HSD post hoc test, *p* < 0.05). Groups 1 and 2 share a superscript *e*, indicating no statistically significant difference between them. Additionally, 95% CI = 95% confidence interval computed as Mean ± (t_0.025, df=7_ × SEM); *n* = 8 per group. NaF = sodium fluoride gel; HANPs = hydroxyapatite nanoparticles; BAGNPs = bioactive glass nanoparticles; ECL = Er, Cr: YSGG laser.

## Data Availability

The data can be made available on request to the authors.
